# Comparing Outcomes of Two Types of Bariatric Surgery in an Adolescent Obese Population: Roux-en-Y Gastric Bypass vs. Sleeve Gastrectomy

**DOI:** 10.3389/fped.2016.00078

**Published:** 2016-07-26

**Authors:** Giovana D. Maffazioli, Fatima Cody Stanford, Karen J. Campoverde Reyes, Takara L. Stanley, Vibha Singhal, Kathleen E. Corey, Janey S. Pratt, Miriam A. Bredella, Madhusmita Misra

**Affiliations:** ^1^Neuroendocrine Unit, Massachusetts General Hospital, Harvard Medical School, Boston, MA, USA; ^2^Pediatric Endocrine Unit, Massachusetts General Hospital for Children, Harvard Medical School, Boston, MA, USA; ^3^Weight Center, Massachusetts General Hospital, Boston, MA, USA; ^4^Department of Medicine-Gastroenterology, Massachusetts General Hospital, Harvard Medical School, Boston, MA, USA; ^5^Department of Surgery, Massachusetts General Hospital, Harvard Medical School, Boston, MA, USA; ^6^Department of Radiology, Massachusetts General Hospital, Harvard Medical School, Boston, MA, USA

**Keywords:** bariatric surgery, adolescence, Roux-en-Y gastric bypass, sleeve gastrectomy, obesity

## Abstract

**Background:**

Obesity is prevalent among adolescents and is associated with serious health consequences. Roux-en-Y Gastric Bypass (RYGB) and Sleeve Gastrectomy (SG) are bariatric procedures that cause significant weight loss in adults and are increasingly being performed in adolescents with morbid obesity. Data comparing outcomes of RYGB vs. SG in this age-group are scarce. This study aims to compare short-term (1–6 months) and longer-term (7–18 months) body mass index (BMI) and biochemical outcomes following RYGB and SG in adolescents/young adults.

**Methods:**

A retrospective study using data extracted from medical records of patients 16–21 years who underwent RYGB or SG between 2012 and 2014 at a tertiary care academic medical center.

**Results:**

Forty-six patients were included in this study: 24 underwent RYGB and 22 underwent SG. Groups did not differ for baseline age, sex, race, or BMI. BMI reductions were significant at 1–6 months and 7–18 months within groups (*p* < 0.0001), but did not differ by surgery type (*p* = 0.65 and 0.09, for 1–6 months and 7–18 months, respectively). Over 7–18 months, within-group improvement in low-density lipoprotein (LDL) (−24 ± 6 in RYGB, *p* = 0.003, vs. −7 ± 9 mg/dl in SG, *p* = 0.50) and non-high-density lipoprotein (non-HDL) cholesterol (−23 ± 8 in RYGB, *p* = 0.02, vs. −12 ± 7 in SG, *p* = 0.18) appeared to be of greater magnitude following RYGB. However, differences between groups did not reach statistical significance. When divided by non-alcoholic steatohepatitis stages (NASH), patients with Stage II–III NASH had greater reductions in alanine aminotransferase levels vs. those with Stage 0–I NASH (−45 ± 18 vs. −9 ± 3, *p* = 0.01) after 7–18 months. RYGB and SG groups did not differ for the magnitude of post-surgical changes in liver enzymes.

**Conclusion:**

RYGB and SG did not differ for the magnitude of BMI reduction across groups, though changes trended higher following RYGB. Further prospective studies are needed to confirm these findings.

## Introduction

Obesity is a major global challenge, which affects children, adolescents, and adults. In the United States, between 2009 and 2010, 31.8% of children aged 2–19 years were classified as overweight and 16.9% as obese (1). Of concern, most children and adolescents with obesity remain obese as adults (2). Obesity causes co-morbidities, such as insulin resistance and type 2 diabetes mellitus (T2D), cardiovascular disease, hyperlipidemia, hypertension, obstructive sleep apnea, non-alcoholic fatty liver disease, polycystic ovary syndrome, and degenerative joint disease (3–5). In addition, obesity is associated with psychiatric conditions, such as depression and eating disorders, and with significant psychosocial stresses, including bullying, isolation, and discrimination. Therefore, reduced quality of life and life expectancy are important consequences of obesity and its related co-morbidities (6).

In this context, there is an urgent need to develop therapies that target obesity in the pediatric and adolescent population. Unfortunately, lifestyle changes, including increased physical activity and dietary modification, achieve limited results (7), and pharmacologic therapy carries the risk of side effects without clear sustained benefit (8). Bariatric surgery is, thus, becoming an alternative for adolescents and young adults with moderate to severe obesity, particularly those with serious co-morbidities, in whom conservative therapy has proven ineffective. Studies have shown important short- and mid-term benefits of weight loss surgery in this population (9), and the American Society for Metabolic and Bariatric Surgery (ASMBS) has proposed that surgery should be offered relatively early to patients with severe obesity, rather than waiting until co-morbidities develop (10, 11).

Roux-en-Y Gastric Bypass (RYGB) and Sleeve Gastrectomy (SG) are the most commonly performed surgical weight loss procedures, and both are well tolerated and effective in adolescents with severe obesity (7, 12, 13). Although the choice of surgical procedure is often based on the patient’s characteristics and physician–patient discussion, data comparing outcomes from different bariatric procedures in the adolescent/young adult population would be useful to better guide surgical choice. A recent study evaluating long-term outcomes of RYGB vs. SG in adolescents showed significant weight reduction following both procedures as well as remission of T2D and dyslipidemia (14). However, impairment in liver function due to non-alcoholic fatty liver disease is also a potential consequence of obesity and it was not verified in the previous study. According to the ESPGHAN Hepatology Committee (15), patients with this condition may benefit from weight reduction following bariatric surgery. The current study aims to add to these data by comparing short-term outcomes at 1–6 months and longer-term outcomes 7–18 months following SG and RYGB performed at a single center with respect to changes in body mass index (BMI), metabolic and lipid profile, and liver enzymes in adolescents with severe obesity.

## Materials and Methods

We conducted a retrospective study with all adolescents, 16–21 years old, who underwent RYGB or SG at our institution between January 2012 and December2014. All patients received pre-operative evaluation and care at the Weight Center of our institution. The choice for RYGB or SG was based on the patient’s preference after a detailed discussion of surgical procedures and outcomes with the surgeon. Data collected include demographics and pre- and post-operative measures of body weight and laboratory tests. Laboratory data include liver enzymes [alkaline phosphatase, alanine aminotransferase (ALT), aspartate aminotransferase (AST)], lipids [total cholesterol, triglycerides, high-density lipoprotein (HDL), low-density lipoprotein (LDL), and calculated non-HDL cholesterol], blood count (hematocrit, hemoglobin, and platelets), creatinine, and calcium metabolism parameters [calcium and 25-hydroxy vitamin D (25-OHD)]. In addition, information regarding a pre-existing diagnosis of T2D was extracted from medical charts. All patients had a liver biopsy at surgery in order to evaluate for non-alcoholic steatohepatitis (NASH). We considered a patient as having T2D if the patient had either a documented pre-operative diagnosis of T2D in the medical charts and either an HbA1c ≥6.5% (16, 17) or was using a hypoglycemic drug (with documented T2D before starting the hypoglycemic drug). Remission of T2D following surgery was defined as a decrease in HbA1c levels to below 6.0% and/or discontinuation of the hypoglycemic drug with subsequent normal fasting and/or random blood glucose. Information regarding duration of surgery, length of hospital stay, and occurrence of post-operative complications was collected.

Data collection was performed using the following criteria: (1) pre-operative laboratory assessments were obtained from a visit within 6 months before surgery. If more than one set of laboratory data was available, the set that was most comprehensive and/or closest to surgery was chosen. Baseline weight measurements were taken within 3 months preceding surgery. Adult height information was also collected. A patient was considered to have attained adult height if height measurements had plateaued on the patient’s growth chart and/or the chronological age at the time of height assessment was at least 15 years in a female and 17 years in a male. This was based on Greulich and Pyle charts (18) that indicate that only 1% of growth persists at bone ages of 15 and 17 in girls and boys, respectively. Of note, children with obesity typically have bone ages that exceed their chronological age (19). Thus, for children with obesity and chronological ages of 15 in girls and 17 in boys, the bone age is likely even greater, indicating completion of statural growth. (2) Weight measurements and laboratory values (i) 1–6 months and (ii) 7–18 months post-operatively were obtained from any clinic visit within the appropriate timeframe. If more than one set of data was available, the most comprehensive set and/or the set farthest from surgery was selected. Post-operative weight measurements were typically available at the time of blood draws. If not, they were obtained from any time point within a month of the blood draw.

All RYGB and SG procedures were performed laparoscopically using a similar technique for all patients undergoing the specific procedure. Eligibility was determined by Weight Center criteria, which for adolescents include a BMI ≥35 kg/m^2^ with severe co-morbidities or BMI ≥40 kg/m^2^ with minor co-morbidities, completion of statural growth, demonstration of previous sustained efforts at non-surgical weight loss (lifestyle changes, including nutritional changes and physical activity), and determination by a physician, psychologist, and dietitian that sufficient maturity exists to recognize risks and benefits of the procedure and implement required post-operative behavioral changes (20). Patients were selected for RYGB or SG based on patient characteristics and the consensus of the Weight Center providers.

This study was approved by our Institutional Review Board, with a waiver of the requirement for informed consent based on the minimal risk for subjects and impracticability of obtaining consent for this retrospective review.

Sample size was based on expected BMI changes after surgery, and was determined based on variance data available from an earlier paper (SD of 3.5 kg/m^2)^ (20). We expected that with 20 patients in each of the surgical groups, the probability was 80% that the study would detect a BMI reduction of at least 3.5 kg/m^2^ over time in each of the groups while keeping the false positive rate to less than 5%.

We used JMP (v10; SAS Institute, Inc., Cary, NC, USA) for all analyses. Data are reported as mean ± SD or median (IQR) (for between-group comparisons), mean difference ± SEM (for within-group changes over time), and 95% confidence intervals. Pre-operative baseline characteristics, and changes in BMI measurements and laboratory values between the two surgery groups were analyzed using the Student *t*-test for normally distributed data or the Wilcoxon rank-sum test for non-normally distributed variables. For the analysis comparing pre- and post-operative measures within groups, we performed a paired *t*-test for normally distributed data and a Wilcoxon signed-rank test for data not normally distributed. Fisher’s exact test was used to compare proportions.

Follow-up information for BMI was available for the RYGB and SG groups at 1–6 months in all subjects and at 7–18 months in 83% of the RYGB and 64% of the SG subjects (*p* = 0.18). Laboratory information at 7–18 months was available in up to 67% of subjects in the RYGB group and 46% of those in the SG group (*p* = 0.23). The use of the paired *t*-test or Wilcoxon signed-rank test mitigates to some extent loss to follow-up as it takes into account only those individuals with data available at baseline (before surgery) and at follow-up. In addition, in our study, patients who were lost to follow-up did not differ from those who returned for follow-up at 7–18 months for (i) gender (2M/10F without follow-up vs. 4M/30F with follow-up, *p* = 0.64), (ii) mean age (19.2 ± 1.7 without follow-up vs. 18.8 ± 1.8 years old with follow-up, *p* = 0.51), (iii) race (0% Asian, 0% Black, 42% Hispanic, and 58% White without follow-up vs. 3% Asian, 9% Black, 33% Hispanic, and 55% White with follow-up, *p* = 0.65), and (iv) BMI at surgery (50.6 ± 8.4 without follow-up vs. 49.8 ± 6.8kg/m^2^ with follow-up, *p* = 0.85). Median follow-up time did not differ between RYGB vs. SG groups [10.7 (7.8–11.8) months vs. 11.2 (8.7–14.6) months, *p* = 0.29].

## Results

### Baseline Characteristics

Forty-six patients underwent weight loss surgery between January 2012 and December 2014; 24 (52%) underwent RYGB and 22 (48%) SG. Of those, 25 patients (54%) were between 16 and 18 years old [14 (56%) in RYGB and 11 (44%) SG groups, *p* = 0.77]. Baseline characteristics are shown in Table 1. Anthropometric measures and pre-operative laboratories were collected over an average period of 4.5 ± 3.9 months prior to surgery for RYGB and 2.7 ± 3.0 months for SG groups with no significant differences between groups (*p* = 0.09). Groups did not differ for age, sex, race, pre-operative weight, or pre-operative BMI. For pre-operative laboratory measurements, patients in the SG group had higher creatinine than RYGB subjects, and patients in the RYGB group had higher LDL than the SG group. Other laboratory measures did not differ by surgery type.

**Table 1:** **Baseline characteristics of groups undergoing SG vs. RYGB procedures**.

	RYGB surgery (*n* = 24)	SG surgery (*n* = 22)	*p*-Value
Age (years)	18.5 ± 1.7 (24)	19.4 ± 1.7 (22)	0.07
Sex (males/females)	1/23	5/17	0.09
Race/ethnicity (%)			0.71
Black	1 (4.2%)	2 (9.1%)	
Hispanic	8 (33.3%)	8 (36.4%)	
White	14 (58.3%)	12 (54.5%)	
Asian	1 (4.2%)	0 (0)	
Weight (kg)	137.2 ± 18.5 (24)	143.7 ± 30.4 (22)	0.72*
BMI (kg/m^2^)	50.3 ± 6.0 (24)	49.7 ± 8.4 (22)	0.79
Liver enzymes			
Alkaline phosphatase (U/l)	78 ± 18 (20)	80 ± 24 (21)	0.94*
ALT (U/l)	30 ± 15 (22)	24 ± 16 (22)	0.09*
AST (U/l)	27 ± 12 (22)	22 ± 8 (22)	0.06*
Lipid profile
Triglycerides (mg/dl)	103 (84–119) (16)	104 (63–141) (10)	1.00*
Total cholesterol (mg/dl)	159 (134–135) (16)	146 (123–168) (10)	0.17
High-density lipoprotein (mg/dl)	39 (33–42) (16)	43 (38–49) (10)	0.18*
Low-density lipoprotein (mg/dl)	101 (79–118) (16)	74 (60–103) (10)	**0.02**
Non-HDL cholesterol (mg/dl)	117 (97–139) (16)	99 (82–123) (10)	0.06*
Blood count
Hematocrit (%)	40 ± 2.5 (18)	39 ± 3.7 (20)	0.22
Hemoglobin (g/dl)	13 ± 1.1 (18)	13 ± 1.2 (20)	0.22
Platelets (th/mm^3^)	311 ± 46 (21)	304 ± 64 (21)	0.67
Serum creatinine (mg/dl)	0.67 ± 0.09 (22)	0.74 ± 0.12 (22)	**0.04**
Calcium (mg/dl)	9.4 ± 0.36 (22)	9.4 ± 0.41 (21)	0.64
25(OH)D (ng/ml)	21 ± 7 (15)	23 ± 15 (12)	0.55
Type 2 diabetes (*n* = 9)	5 out of 24	4 out of 22	1.00
Non-alcoholic steatohepatitis (*n* = 14)^a^	8 out of 24	6 out of 22	0.75

*Data are presented as mean ± SD or median (IQR). Sample sizes for each measure are shown in parentheses*.

*The Wilcoxon rank-sum test was used to compare variables that were not normally distributed.

*^a^Non-alcoholic steatohepatitis was diagnosed histologically by liver biopsy at the time of surgery*.

*BMI, body mass index; ALT, alanine aminotransferase; AST, aspartate aminotransferase; 25(OH)D, 25-hydroxyvitamin D*.

*Normative ranges: ALP (15–350 U/l), ALT (7–33 U/l), ASP (9–32 U/l), triglycerides (40–150 mg/dl), total cholesterol (<200 mg/dl), high-density lipoprotein (35–100 mg/dl), low-density lipoprotein (<130 mg/dl), non-HDL cholesterol (<140 mg/dl), hematocrit (37–49%, males; 36–46%, females), hemoglobin (13–16 g/dl, males; 12–16 g/dl, females), platelets (150–450 th/mm^3^), calcium (8.5–10.5 mg/dl), 25(OH)D (33–96 ng/ml)*.

*Bold indicates that values are statistically significant*.

### Changes in Weight and BMI

In the immediate post-operative period (1–6 months post-operatively), BMI decreased significantly from baseline in both RYGB (−7.9 ± 0.7kg/m^2^, *p* < 0.0001) and SG (−7.5 ± 0.6 kg/m^2^, *p* < 0.0001) groups compared to pre-operative values. These changes were not significantly different between groups (*p* = 0.65). As shown in Table 2 for outcomes assessed after 7–18 months of follow-up, there was again a marked reduction in BMI for both RYGB (−15.9 ± 1.1 kg/m^2^, *p* < 0.0001) and SG (−12.6 ± 1.7 kg/m^2^, *p* = 0.0002) groups, with a trend toward greater reductions in the RYGB group (*p* = 0.09). Percentage of excess BMI lost (%EBMIL) and percentage of total body weight loss did not differ between the RYGB and SG groups (Table 2). Only one patient had weight regain following SG (8.2 kg), while no patient regained weight after RYGB over an 18-month period. Weight regain was not seen in any subject at 1–6 months following surgery.

**Table 2:** **Changes in BMI and biochemical parameters in RYGB and SG groups 7–18 months following surgery**.

	RYGB surgery				SG surgery				*P* (LSG vs. RYGB)
	Pre-operative	7–18 months post-operative	Mean difference	Paired *t*-test	Pre-operative	7–18 months post-operative	Mean difference	Paired *t*-test	
	
	Mean ± SD	Mean ± SD	Mean ± SEM	*P*	Mean ± SD	Mean ± SD	Mean ± SEM	*P*	
BMI (kg/m^2^)	50.6 ± 6.5 (20)	34.7 ± 5.7 (20)	−15.9 ± 1.1	**<0.0001**	48.7 ± 7.4 (14)	36.1 ± 11.3 (14)	−12.6 ± 1.7	0.0002*	0.09
% Excess BMI lost	–	–	−63.4 ± 4.7	–	–	–	−58.4 ± 5.6	–	0.50
% Total body weight loss	–	–	−31.3 ± 2.2	–	–	–	−26.8 ± 2.7	–	0.20
ALP (U/l)	81 ± 18 (14)	86 ± 28 (14)	5 ± 5	0.29	80.1 ± 29 (10)	73 ± 19 (10)	−7 ± 7	0.31	0.13
ALT (U/l)	32 ± 16 (16)	23 ± 20 (16)	−9 ± 5	0.03*	29 ± 22 (10)	17 ± 10 (10)	−11 ± 7	0.08*	0.56*
AST (U/l)	28 ± 12 (16)	23 ± 14 (16)	−5 ± 4	0.14*	24 ± 11 (10)	19 ± 5 (10)	−6 ± 4	0.11*	0.87*
Triglycerides (mg/dl)	96 ± 18 (9)	101 ± 62 (9)	5 ± 21	0.68*	117 ± 40 (4)	90 ± 39 (4)	−27 ± 16	0.18	0.40*
Total Cholesterol (mg/dl)	164 ± 21 (9)	148 ± 23 (9)	−16 ± 5	0.02*	152 ± 24 (4)	150 ± 18 (4)	−2 ± 8	0.77	0.19
HDL (mg/dl)	39 ± 3 (9)	46 ± 13 (9)	7 ± 4	0.10	43 ± 11 (4)	53 ± 13 (4)	10 ± 3	0.06	0.73
LDL (mg/dl)	106 ± 18 (9)	82 ± 15 (9)	−24 ± 6	**0.003**	86 ± 25 (4)	79 ± 13 (4)	−7 ± 9	0.50	0.12
Non-HDL (mg/dl)	125 ± 20 (9)	102 ± 27 (9)	−23 ± 8	0.02*	109 ± 31 (4)	97 ± 19 (4)	−12 ± 7	0.18	0.40
Hematocrit (%)	40.5 ± 2.8 (11)	38.8 ± 2.9 (11)	−2 ± 1	0.14	39.9 ± 3.8 (10)	39.9 ± 3.9 (10)	0.0 ± 0.5	0.97	0.18
Hemoglobin (g/dl)	13.4 ± 1.3 (11)	12.7 ± 1.2 (11)	−0.7 ± 0.4	0.10	13.1 ± 1.3 (10)	13.2 ± 1.3 (10)	0.1 ± 0.2	0.64	0.09
Platelets (th/mm^3^)	309 ± 51 (15)	267 ± 56 (15)	−42 ± 15	**0.01**	283 ± 78 (10)	254 ± 55 (10)	−30 ± 11	**0.02**	0.56
Creatinine (mg/dl)	0.7 ± 0.1 (16)	0.7 ± 0.1 (16)	0.0 ± 0.0	0.37*	0.7 ± 0.1 (10)	0.7 ± 0.1 (10)	0.0 ± 0.0	**0.02**	0.67
Calcium (mg/dl)	9.5 ± 0.4 (16)	9.5 ± 0.4 (16)	0.0 ± 0.1	0.67	9.4 ± 0.5 (10)	9.5 ± 0.5 (10)	0.1 ± 0.1	0.51	0.81
25(OH)D (ng/ml)	21 ± 8 (9)	30 ± 13 (9)	9 ± 5	0.13	27 ± 19 (7)	35 ± 18 (7)	8 ± 5	0.16	0.92*

*Sample size for each measure is shown in parentheses*.

*Wilcoxon signed-rank test was used for non-normally distributed data.

*BMI, body mass index; ALP, alkaline phosphatase; ALT, alanine aminotransferase; AST, aspartate aminotransferase; HDL, high-density lipoprotein cholesterol; LDL, low-density lipoprotein cholesterol; 25(OH)D, 25-hydroxyvitamin D*.

*Normative ranges: ALP (15–350 U/l), ALT (7–33 U/l), ASP (9–32 U/l), triglycerides (40–150 mg/dl), total cholesterol (<200 mg/dl), HDL (35–100 mg/dl), LDL (<130 mg/dl), non-HDL (<140 mg/dl), hematocrit (37–49%, males; 36–46%, females), hemoglobin (13–16 g/dl, males; 12–16 g/dl, females), platelets (150–450 th/mm^3^), calcium (8.5–10.5 mg/dl), 25(OH)D (33–96 ng/ml)*.

*Bold indicates that values are statistically significant*.

### Changes in Diabetes Status

There were nine cases of pre-existing T2D, five in the RYGB group and four in the SG group. Overall, six patients had remission of T2D. Of the five pre-existing cases in the RYGB group, two patients had remission of T2D, one had normal HbA1c levels on a hypoglycemic drug, one had improvement in glucose levels but was not able to discontinue oral hypoglycemics, and the other was lost to follow-up. Of the four pre-existing cases in the SG group, three had initial remission of T2D, though one of these three patients had weight regain during follow-up with recurrence of diabetes. The fourth patient in the SG group had improvement in glucose levels but continued to need diabetes medication.

### Changes in Liver Enzymes

The RYGB group had significantly greater reductions in ALT (*p* = 0.03) but not in AST levels 7–18 months post-operatively, whereas the SG group had a trend toward a reduction in ALT (*p* = 0.08) but not in AST over the follow-up period. NASH, diagnosed by liver biopsy at the time of surgery, was present in 14 of 46 patients (8 in the RYGB and 6 in the SG groups). Subjects with evidence of NASH were classified according to NASH Stages (21). Forty-three percent (*n* = 6) had Stage I, 14% (*n* = 2) Stage II, and 14% (*n* = 2) Stage III NASH. No patient was diagnosed with Stage IV disease. Twenty-nine percent (*n* = 4) of patients with NASH had no fibrosis. When we divided patients into two categories according to NASH stages (Stages 0–I and II–III), we found that compared to patients with Stage 0–I NASH, those with Stage II–III NASH had greater baseline values for ALT (29 ± 8 vs. 67 ± 25, *p* = 0.001) and AST (25 ± 13 vs. 47 ± 23, *p* = 0.046). At 1–6 months follow-up, patients with Stage II–III NASH had greater decreases in ALT (−45 ± 18 vs. −10 ± 3, *p* = 0.02) but not in AST (−19 ± 27 vs. −2 ± 2, *p* = 0.33) compared with patients with Stage 0−I NASH. Similarly, at 7−18 months follow-up, there were significant reductions in ALT (−45 ± 18 vs. −9 ± 3, *p* = 0.01) but not AST (2 ± 2 vs. −5 ± 2, *p* = 0.32) in those with Stage II−III NASH vs. those with Stage 0−I NASH. RYGB and SG groups did not differ for the magnitude of post-surgical changes in liver enzymes.

### Changes in Lipids

Nine patients in the RYGB group and four patients in the SG group had dyslipidemia at baseline. This difference was not statistically significant between groups, *p* = 0.23. The RYGB group had significant reductions in LDL (*p* = 0.003) and non-HDL cholesterol (*p* = 0.02), but not HDL cholesterol at 7–18 months follow-up (Table 2). The SG group did not have significant changes in the lipid profile, although there was a trend toward an increase in HDL cholesterol (*p* = 0.06). Between-group comparisons of changes in lipids over 7–18 months did not show significant differences, even after controlling for baseline values.

### Changes in Blood Count Parameters

Changes in hematocrit and hemoglobin levels over the follow-up period did not differ either within groups or between groups undergoing RYGB or SG (Table 2). Platelets decreased in both RYGB and SG groups (*p* = 0.01 and 0.02, respectively) (Table 2).

### Changes in Calcium Metabolism

Calcium and 25(OH) D levels did not change in either group over 7–18 months. Groups did not differ for prevalence of vitamin D deficiency either before or after surgery (data not shown).

### Surgical Time and Post-Operative Complications

Mean surgical time for RYGB vs. SG, respectively, was 3.1 ± 0.3 vs. 2.6 ± 0.5 h (*p* = 0.001), and mean length of hospital stay was 2.2 ± 0.4 vs.1.9 ± 0.3 days (*p* = 0.01). Post-operative complications occurred in one patient following RYGB (post-prandial vomiting) and no patient following SG (*p* = 0.33).

## Discussion

Few data are currently available comparing outcomes of different bariatric surgery techniques in the adolescents. Our data demonstrate 1–6 months and 7–18 months reductions in BMI following both RYGB and SG, and no difference between the groups for changes in BMI and weight regain following surgery, indicating that both RYGB and SG are effective options for weight loss in adolescents. This is consistent with prior studies by Inge et al. (14) and Cozacov et al. (22). However, weight reduction at 7–18 months following RYGB trended higher than that following SG. Although surgical time and length of stay at the hospital were greater in the RYGB group, the rate of post-operative complications did not significantly differ between groups. This is in accordance with prior studies (23) and supports the safety profile of both procedures.

Thirty percent of patients had findings consistent with NASH on liver biopsy performed at the time of surgery. Following surgery, transaminases improved in both groups, suggesting improvement in fatty liver disease, although this was not verified by repeat biopsy. A decrease in ALT occurred following both RYGB and SG. When considering patients who had a diagnosis of NASH at the time of surgery, patients presenting with Stages II–III had greater improvement in their liver enzymes when comparing to those classified as Stages 0–I. This is consistent with our previous study, which showed a decrease in AST and ALT following RYGB in a subgroup of patients who had elevated baseline liver function tests, but not in the whole group of patients (20). Moreover, Xanthakos et al. (24) found a positive association between higher ALT levels and chances of more severe non-alcoholic fatty liver disease. To our knowledge, data are not available regarding changes in AST/ALT following SG in adolescents, and our study indicates no differences between RYGB vs. SG groups for changes in liver enzymes.

With regard to metabolic parameters, lipid levels improved following both RYGB and SG. Seven to 18 months changes in LDL appeared to be of greater magnitude in the RYGB group. These findings suggest that RYGB may prove to be more beneficial for LDL improvement when compared to SG and may be favored in those with severe dyslipidemia. However, baseline LDL values were higher in the RYGB group, and we cannot exclude regression to the mean as an explanation for this finding. The improvement in lipid levels seen in our study is consistent with previous literature (7, 13, 14, 20). However, changes in lipid values were at the normal range and the clinical impact of these is still poorly understood. Further evaluation of the long-term impact of RYGB on lipids and cardiovascular disease outcomes is needed.

Anemia has been reported in 25–50% of the patients following RYGB (25, 26). Iron deficiency anemia is the most common cause and it may take years to develop. In our study, anemia was not observed during the 7–18 months follow-up period in either of the groups. This may be a reflection of a relative short follow-up period and/or an increased intake of supplements post-operatively.

Although vitamin D deficiency is common following bariatric surgery (27) and reported in 50–80% patients after RYGB (28), our study demonstrated no decrease in vitamin D levels for either surgical group over 7–18 months of follow-up. This may be a reflection of increased supplement intake after surgery and/or a high prevalence of vitamin D insufficiency prior to surgery. Vitamin D regulates calcium metabolism through its impact on PTH secretion, and our patients demonstrated normal PTH levels at 7–18 months follow-up with no difference across groups.

Our study has some limitations. First, this is a retrospective study and uses clinical data that were not uniformly collected in all subjects. It is possible that subjects with more favorable outcomes are more likely to present for clinical follow-up, whereas those without favorable outcomes are less likely to return for follow-up. However, it is also possible that those with the best outcome no longer see a reason to follow up and are the ones that are lost follow-up. A second limitation is that patients who underwent RYGB and SG were not matched for some baseline measures, such as creatinine and LDL, which may have influenced some results of comparisons of post-surgical changes. In addition, we have a relatively small sample size and a limited time frame for evaluation of post-operative changes. Longer studies with larger sample sizes will be needed to confirm the present findings. Finally, we did not have sufficient post-operative fasting glucose and insulin values available to report quantitative changes in glucose metabolism. However, in evaluating patients with pre-existing diabetes, we found that at least 67% of subjects had achieved remission and 22% had improved glycemic levels following surgery. This is consistent with previous studies showing remission of T2D following bariatric procedures (7, 12–14, 20, 22).

In spite of its limitations, the current study adds to the literature, as there are limited data regarding outcomes of bariatric surgery in adolescents and young adults and there is only one larger study comparing outcomes of RYGB vs. SG in this population (14). However, the prior study did not report effects of surgery on liver enzymes. Consistent with this prior study, we demonstrate no difference in changes in BMI when comparing procedures. Further studies are also necessary to better understand differences in laboratory outcomes and resolution of co-morbidities between the two procedures.

## Author Contributions

All authors have made substantial contribution to the paper: GM and FS were responsible for drafting the manuscript and interpreting the data; KR was responsible for acquisition of data and data analysis; TS and MM were responsible for the conception and design of the study and critical review of the paper. VS, KC, JP, and MB were responsible for revising the article critically and adding important intellectual content. All authors have read and approved the final version of the paper.

## Conflict of Interest Statement

The authors declare that the research was conducted in the absence of any commercial or financial relationships that could be construed as a potential conflict of interest.
